# Risk factors for pulmonary hypertension in patients receiving maintenance peritoneal dialysis

**DOI:** 10.1590/1414-431X20154733

**Published:** 2016-02-02

**Authors:** Y. Zeng, D.D. Yang, S. Feng, H.Y. Shen, Z. Wang, S. Jiang, Y.B. Shi, J.X. Fu

**Affiliations:** 1 Department of Nephrology, The Second Affiliated Hospital of Soochow University, Suzhou, China; 2 Department of Hematology, The Second Affiliated Hospital of Soochow University, Suzhou, China

**Keywords:** Maintenance peritoneal dialysis, Pulmonary hypertension, End-stage renal disease, Internal arteriovenous fistula, Hypertensive nephropathy, Ejection fraction

## Abstract

We investigated the risk factors for pulmonary hypertension (PH) in patients receiving maintenance peritoneal dialysis (MPD). A group of 180 end-stage renal disease patients (124 men and 56 women; mean age: 56.43±8.36) were enrolled in our study, which was conducted between January 2009 and June 2014. All of the patients received MPD treatment in the Dialysis Center of the Second Affiliated Hospital of Soochow University. Clinical data, laboratory indices, and echocardiographic data from these patients were collected, and follow-ups were scheduled bi-monthly. The incidence and relevant risk factors of PH were analyzed. The differences in measurement data were compared by t-test and enumeration data were compared with the χ2 test. Among the 180 patients receiving MPD, 60 were diagnosed with PH. The remaining 120 were regarded as the non-PH group. Significant differences were observed in the clinical data, laboratory indices, and echocardiographic data between the PH and non-PH patients (all P<0.05). Furthermore, hypertensive nephropathy patients on MPD showed a significantly higher incidence of PH compared with non-hypertensive nephropathy patients (P<0.05). Logistic regression analysis showed that the proportion of internal arteriovenous fistula, C-reactive protein levels, and ejection fraction were the highest risk factors for PH in patients receiving MPD. Our study shows that there is a high incidence of PH in patients receiving MPD and hypertensive nephropathy patients have an increased susceptibility to PH.

## Introduction

End-stage renal disease (ESRD) affects 10-16% of adults worldwide. It is characterized by a significantly reduced estimated glomerular filtration rate and increased urinary albumin excretion (1). ESRD is clinically defined as kidney failure requiring dialysis or transplantation, and is associated with high healthcare costs and mortality. ESRD costs nearly US$23 billion each year in health care costs in the US, and the mortality rates are eight times higher in 20- to 64-year-old ESRD patients treated by dialysis than those individuals of similar age (2). The United States Renal Data System annual data report showed that the mortality of chronic kidney disease (CKD) patients in 2008 was 1.7 times higher than that of non-CKD patients after adjusting for age, gender, race, prior hospitalizations, and comorbidity (3). In-center hemodialysis and home peritoneal dialysis (PD) are the two most common dialysis therapies. Costs associated with PD are almost US$20,000 lower than those of hemodialysis, but PD and hemodialysis have similar health outcomes (4). Maintenance peritoneal dialysis (MPD) is a common renal replacement therapy for ESRD patients (3). However, MPD is complicated by diseases such as pulmonary hypertension (PH).

PH was previously classified into two categories, primary PH or secondary PH, based on the presence of identified causes or risk factors (5). PH is linked to diverse etiologies, such as left heart failure, chronic hypoxic lung diseases, collagen vascular disease, portal hypertension, chronic recurrent thromboembolism, human immunodeficiency virus infection, and exposure to drugs and toxins (6 7 8). PH is frequently associated with CKD and ESRD, and is hemodynamically defined as a mean pulmonary artery pressure (PAP) greater than 25 mmHg. A recent study suggested that left heart dysfunction is the underlying cause of PH in patients with kidney disease (9). The incidence of PH is high in ESRD patients and ranges from 9 to 39% in Stage 5 CKD patients, between 18.8 and 68.8% in hemodialysis patients, and between 0 and 42% in patients receiving PD treatment (10). Several mechanisms have been proposed for the incidence of PH in kidney diseases, such as left ventricular disorders, arteriovenous fistula, volume overload, sleep disorder, dialysis membrane exposure, endothelial dysfunction, and vascular calcification (11). Genctoy et al. found that older age, lower ejection fraction, and secondary hyperparathyroidism may contribute to PH in CKD with proteinuria (12). Alhamad et al. studied the clinical characteristics of PH patients receiving hemodialysis or peritoneal dialysis versus patients receiving renal transplantation (13). However, few studies have investigated the incidence of PH in patients receiving MPD and the risk factors promoting PH in MPD patients.

In this study, we investigated the incidence of PH in patients receiving MPD. We also examined potential risk factors through a detailed analysis of clinical data, laboratory indices, and echocardiographic data that were collected during bi-monthly follow-up visits.

## Material and Methods

### Patients

Between January 2009 and June 2014, 180 ESRD patients receiving MPD treatment at the Dialysis Center of the Second Affiliated Hospital of Soochow University were selected for this study, which was approved by the Hospital’s Ethical Committee. The patient group consisted of 124 men and 56 women (mean age: 56.43±8.36; mean dialysis time: 36.35±3.38 months). Inclusion criteria for patients in this study were as follows: *i* ) patients were treated by continuous ambulatory peritoneal dialysis and their daily cumulative dialysate dose was 6-8 L; *ii* ) patients received renal replacement therapy for longer than 6 months and were in a stable condition; *iii* ) patients were compliant and willing to be followed up; and *iv* ) patients were older than 18 years of age. All of the patients received pulmonary perfusion imaging and showed even perfusion without perfusion defects. Written informed consent was obtained from all patients.

The exclusion criteria of patients were as follows: *i* ) patients with chronic lung disease that could greatly affect PAP (14) (e.g., dyspnea and a state of air intake with arterial blood gas analysis showing a pH <7.35, chronic thromboembolic disease, which can also greatly affect PAP (15), obvious arteriovenous ischemia, or thromboembolism diagnosed by imaging methodology); *ii* ) patients having received renal replacement therapy within the past 6 months or who were hospitalized during renal replacement therapy within the past 3 months; *iii* ) patients with severe left heart disease, such as New York Heart Association class III/IV heart failure and a left ventricular ejection fraction <50%; and *iv* ) patients having received prior hemodialysis. Additionally, we excluded patients with the following diseases that cause pulmonary hypertension: congenital heart disease, acute coronary syndrome, postinfarction syndrome, valvular heart disease (valvular regurgitation or grade II or above valvular stenosis), pericardial disease, autoimmune disease, chest wall or lung parenchymal disease, and pulmonary embolism.

### Collection of clinical data, laboratory indices, and echocardiographic data

Clinical data including age, gender, smoking (ex-smokers or current smokers), height, weight, interdialytic weight gain, total dialysate, average ultrafiltration volume, liquid removal volume, pulmonary function tests (forced vital capacity and forced expiratory volume in 1 second), dialysis time, systolic pressure, diastolic pressure, mean arterial pressure, and body mass index were recorded. Chronic renal failure and its complications were also recorded. Laboratory indices were recorded, including the serum levels of albumin, total bilirubin, alanine transaminase, aspartate aminotransferase, hemoglobin, brain natriuretic peptide (BNP), parathyroid hormone, calcium, phosphorus, creatinine, urea nitrogen, and C-reactive protein (CRP). Ultrasonic cardiographic data were recorded, including the right ventricular diameter, right ventricular outflow tract diameter, main pulmonary artery diameter, left atrial diameter, left ventricular diastolic diameter, left ventricular systolic diameter, left ventricular outflow tract diameter, interventricular septal thickness, aortic root dimension, ejection fraction, mitral regurgitation, pericardial effusion, and left ventricular mass index.

### Follow-up and diagnostic criteria of PH

After collecting clinical data, laboratory indices and data from ultrasonic cardiograms were collected. Patients were followed up bi-monthly. The termination of follow-up included patients with complicated PH or a follow-up time of longer than 36 months. During follow-up, PAP was evaluated by echocardiography. Systolic tricuspid regurgitant jet velocity (V) was measured directly, and PAP was calculated according to Bernoulli’s equation: PAP = 4 × V^2^ + 10 mmHg (16,17). PH was confirmed by a systolic PAP ≥35 mmHg according to echocardiographic assessment of the right heart guidelines published by the American Society of Echocardiography in 2010 (18). The date and time of diagnosis of PH were recorded, and patients were then categorized into either the PH group or the non-PH group. The clinical differences between PH and non-PH groups were compared to identify risk factors for PH in patients receiving MPD.

### Statistical analysis

Data were analyzed by SPSS 20.0 (IBM Corp., USA). Measurement data are reported as means±SD and the differences were measured by the *t* -test. Enumeration data are reported as percentage or rate, and the differences were compared with the χ^2^ test. Factors related to PH were analyzed by logistic regression analysis. A P value <0.05 was considered to be significant.

## Results

### Patients receiving MPD diagnosed with PH

After examination of the 180 patients by echocardiography, 60 were diagnosed with PH and categorized into the PH group, with PAP ranging from 35.0 to 62.2 mmHg (mean PAP: 53.6±9.6 mmHg). Therefore, the incidence of PH in this cohort was 33.3%. In the PH group, 40 patients were men, 20 were women, and the mean age was 59.65±11.24 years. The remaining 120 patients were categorized as non-PH and included 84 men and 36 women, with a mean age of 61.15±10.61 years. PAP of the non-PH patients ranged from 12.5 to 34.8 mmHg (mean PAP: 21.3±6.4 mmHg).

### Clinical data

Smoking, systolic pressure, diastolic pressure, mean arterial pressure, and the proportion of internal arteriovenous fistula were significantly higher in the PH group than in the non-PH group (all P*<* 0.05). However, there were no significant differences in age, gender, body mass index, interdialytic weight gain, dialysis time, total dialysate, average ultrafiltration volume, liquid removal volume, forced vital capacity, and forced expiratory volume in 1 second between the PH and the non-PH groups (all P>0.05; Table 1).

**Table:**
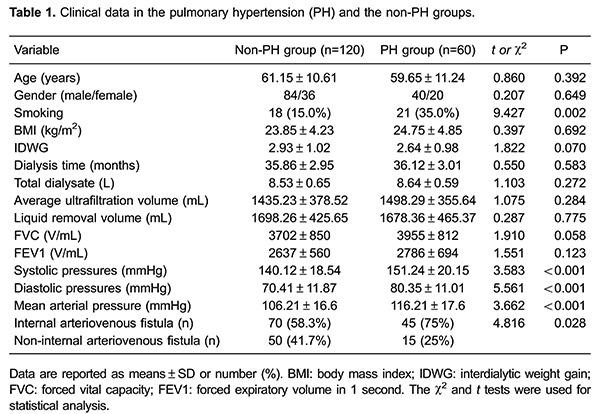

### Laboratory indices

Serum BNP, phosphorus, and CRP levels were significantly higher in the PH group than in the non-PH group (all P*<* 0.05). However, serum albumin levels and hemoglobin were significantly lower in the PH group than in the non-PH group (P*<* 0.05). There were no significant differences in serum total bilirubin, aspartate aminotransferase, alanine transaminase, parathyroid hormone, calcium, creatinine, and urea nitrogen levels between the two groups (P>0.05; Table 2).

**Table:**
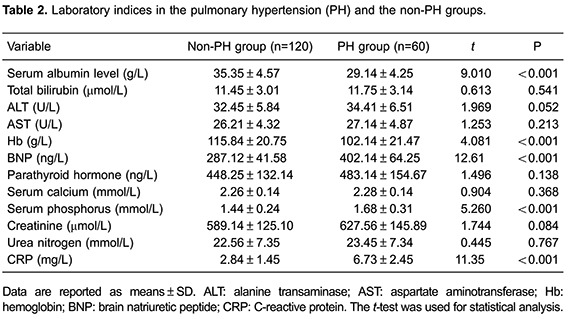

### Primary disease

With regard to primary diseases in the 180 patients receiving MPD, 35% had chronic glomerulonephritis, 33.3% had hypertensive nephropathy, 18.9% had diabetic nephropathy, and 12.8% had other diseases, such as aristolochic acid nephropathy or polycystic kidney disease The incidence of PH was significantly higher in hypertensive nephropathy patients than in any of the non-hypertensive nephropathy patients (P<0.05). There was no significant difference in the incidence of PH between chronic glomerulonephritis patients, diabetic nephropathy patients, or other primary disease patients (P>0.05; Table 3), indicating that these diseases had a minimal influence on PH in MBD patients.

**Table:**
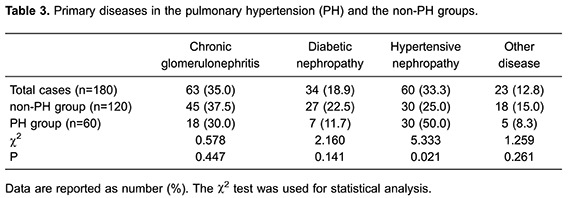

### Echocardiography

The PH group showed a significantly higher right ventricular diameter, right ventricular outflow tract diameter, main pulmonary artery diameter, left atrial diameter, and interventricular septum thickness compared with the non-PH group (all P*<* 0.001). The incidence of mitral regurgitation and pericardial effusion was also significantly higher in the PH group compared with the non-PH group (both P*<* 0.05). However, the ejection fraction was significantly lower in the PH group compared with the non-PH group (P*<* 0.01). No significant changes in left ventricular diastolic diameter, left ventricular outflow tract, and aortic root dimension were observed between the PH and non-PH groups (all P>0.05). The left ventricular mass index was also not significantly different between the two groups (both P>0.05; Table 4).

**Table:**
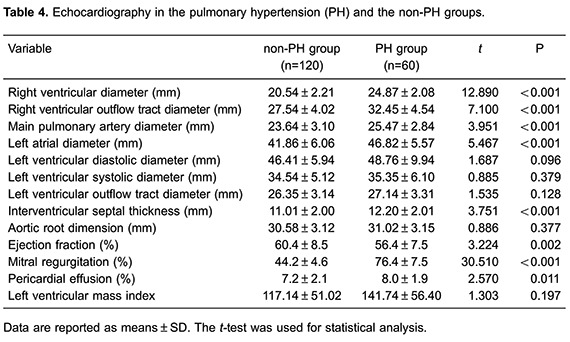

### Analysis of risk factors for patients receiving MPD complicated by PH

We next examined the risk factors for development of PH using logistic regression analysis of systolic blood pressure, diastolic blood pressure, mean arterial pressure, the proportion of arteriovenous fistula, serum BNP levels, phosphorus levels, CRP levels, albumin levels, hemoglobin levels, hypertensive nephropathy, right ventricular diameter, right ventricular outflow tract diameter, main pulmonary artery, left atrial diameter, interventricular septum thickness, ejection fraction, mitral regurgitation, and pericardial effusion. These factors were found to be significantly different in single factor analysis when comparing the PH group with the non-PH group. A forward conditional logistic regression method was used and showed that the proportion of arteriovenous fistula, CRP levels, and ejection fraction were the highest risk factors for PH in patients receiving MPD (Table 5).

**Table:**
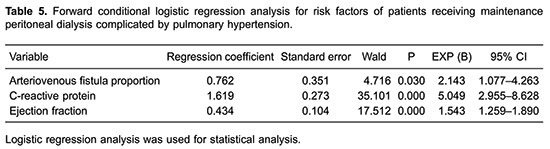

## Discussion

This is the first prospective study to analyze patients receiving MPD for complications leading to PH. We found a high occurrence of PH (33.3%) in this cohort of ESRD patients receiving MPD, which is consistent with previous studies reporting similar high incidence rates. One previous study observed a prevalence of PH as high as 30-60% in cohorts and correlated this high incidence with increased mortality and poor renal transplantation outcome (19). Another study showed that uremic patients receiving chronic dialysis treatment had a high prevalence of PH. This effect was mainly due to elevated pulmonary blood flow and increased pulmonary vascular resistance, which was worsened by fluid overload in hemodialysis patients (20). PH in CKD patients may be induced by left ventricular disorders and some of the typical risk factors of CKD (21). Leung et al. also demonstrated that variation in PH is largely explained by the degree of pulmonary venous hypertension (22). The pathogenesis of PH in ESRD patients might also be due to alterations in endothelial function, increased cardiac output, and myocardial dysfunction, leading to an elevated left heart filling pressure (9).

To examine the underlying cause of PH in patients receiving MPD, we analyzed clinical data, laboratory indices, and echocardiographic data. We also used echocardiography in the PH and non-PH groups during follow-up visits. We found that smoking, systolic pressure, diastolic pressure, mean arterial pressure and the proportion of internal arteriovenous fistula were significantly higher in the PH group than in the non-PH group. Smoking remains a significant risk factor for lung disease and is also related to PH (23,24). Estimated systolic pressure and mean pulmonary arterial pressure are the two main echocardiographic variables used to evaluate PH patients (25). PH is defined as a mean PAP of at least 25 mmHg at rest (19). Internal arteriovenous fistula remains the best choice for vascular access in hemodialysis patients (26). After successfully creating an arteriovenous fistula, arterial blood flow increases 10- to 20-fold, which is associated with arterial and venous dilation, possibly leading to PH (27).

In the current study, laboratory indices showed that BNP, serum phosphorus, and CRP levels were significantly higher in the PH group than in the non-PH group. This finding suggests that these factors might be involved in the development of PH in patients receiving MPD. BNP released from ventricular myocytes is a powerful predictor in cardiovascular disease patients with atrial fibrillation, correlating with higher post-stroke mortality and cardio-embolic stroke (28). Additionally, elevated serum BNP levels are also reported in acute pulmonary embolism patients (29). Plasma BNP has also been reported by many studies as a prognostic indicator in patients with primary PH due to some degree of right ventricular dysfunction (30,31). Serum phosphorus levels play an important role in energy production, membrane transport, and signal transduction (32). However, a report showed no significant differences in serum phosphorus between hemodialysis and PH (33). CRP has pro-inflammatory and anti-inflammatory actions, and its pro-inflammatory effects include induction of inflammatory cytokines and tissue factor. The level of CRP correlates with PH and is an independent predictor of PH (34), as observed in this study.

In the current study, the incidence of PH in hypertensive nephropathy patients was significantly higher than that in non-hypertensive nephropathy patients, indicating that hypertensive nephropathy could lead to the development of PH. Hypertensive nephropathy associated with a progressive decline in glomerular filtration rate, despite reduced blood pressure (35), is the second leading cause of complete kidney failure. This condition is associated with significant morbidity and mortality (36).

In our study, based on echocardiography results, the right ventricular diameter, right ventricular outflow tract diameter, main pulmonary artery diameter, left atrial diameter, and interventricular septum thickness were significantly higher in the PH group compared with the non-PH group, along with a significantly higher incidence of mitral regurgitation and pericardial effusion in the PH group. However, the ejection fraction was significantly lower in the PH group than in the non-PH group. PH is closely linked with right ventricular dysfunction and left ventricular systolic dysfunction, as well as left-sided valvular disease with chronic elevation in left ventricular filling pressure (37). Ventricular hypertrophy, such as increased ventricular diameter, occurs in response to pressure overload in PH (38). Our results of logistic regression analysis further confirmed that the proportion of arteriovenous fistula, CRP levels, and ejection fraction are the highest risk factors for PH in patients receiving MPD.

There are some limitations in the present study. First, we used an echocardiogram for the diagnosis of PH, which is not as accurate as right cardiac catheterization (39). PAP is conventionally evaluated by a right cardiac catheterization procedure. However, in our study, PAP was evaluated by echocardiogram because of the need to perform repeated PAP evaluations over an extended period of time. Additionally, the right cardiac catheterization procedure is traumatic and might affect the patient's compliance with examinations. Second, we used laboratory indices, such as BNP and serum phosphorus, to analyze the potential mechanism of PH in patients receiving MPD. BNP is mainly used as a prognostic indicator for PH patients. The relationship between serum phosphorus and the development of PH is controversial.

Taken together, our results provide an important clinical reference for the risk of PH in patients receiving MPD. Further investigation is likely to reveal early diagnostic and treatment methods in patients receiving MPD, which are urgently needed, as well as the precise mechanisms of development of PH in patients receiving MPD.
